# The implementation of speckle tracking echocardiography for cardiac resynchronization therapy optimisation. A rotational myocardial mechanics interpretation

**DOI:** 10.1186/s44156-024-00062-z

**Published:** 2024-12-02

**Authors:** Alexandros Stefanidis, Paraskevi Korlou, Panagiotis Margos, Ignatios Ikonomidis, Ioannis Paraskevaidis, Konstantinos Gatzoulis, Evmorfia Aivalioti, Konstantinos Kostopoulos

**Affiliations:** 1 1st Cardiology Department, General Hospital of Nikea, Agios Panteleimon, 3 D. Mantouvalou str., 18454 Athens, Greece; 2 Division of Clinical Therapeutics, Alexandra Regional Hospital, Athens, Greece; 3 2nd Cardiology Department, Attikon Hospital of the National and Kapodistrian University of Athens, Athens, Greece; 4 1st Cardiology Department, Hippokration Hospital of the National and Kapodistrian University of Athens, Athens, Greece

**Keywords:** Resynchronization therapy, Rotational mechanics, Speckle tracking echocardiography

## Abstract

**Background:**

Cardiac resynchronization therapy (CRT) has an additive therapeutic influence on left ventricular function in heart failure patients, but the underlying mechanisms through which it works are not completely explained. Our aim was to further elucidate the role of this intervention via rotational mechanics using 2D speckle tracking echocardiography (2D-STE).

**Results:**

We investigated 46 patients (65 ± 9 years) who received CRT. All enrolled patients were assessed on admission by 2D-STE and 6 min walk test (6 min WT) and followed in the outpatient device clinic by 2D-STE (at 1 week and 6 months post-implantation) and 6 min WT (at 6 months post-implantation). On their first appointment all biventricular systems were optimised by atrioventricular delay optimisation and by changing the temporal activation of ventricular electrodes aiming to reach the highest left ventricular effective stroke volume across all activation options. A new 2D-STE based index (twist integral) targeting to assess the rotational mechanics of the whole cardiac cycle was also measured to further explain the CRT response. Twenty-two (48%) patients were responders at 6-month follow-up and most of them had dilated cardiomyopathy. The commonest selected mode that was related with the greatest left ventricular performance response was the simultaneous activation of the 2 ventricular leads (39%). The strongest predictor of CRT response was the improvement of effective stroke volume between admission and first appointment at clinic, followed by the improvement of twist integral, the absence of coronary artery disease, and the improvement of peak systolic twist.

**Conclusions:**

Additional CRT optimisation via changing the temporal activation of ventricular electrodes is beneficial for left ventricular performance in heart failure patients. The success of biventricular pacing may also be explained by the improvement of left ventricular rotational mechanics.

## Background

Cardiac resynchronization therapy (CRT) is currently an established treatment for heart failure (HF) patients with impaired left ventricular ejection fraction (LVEF) and wide QRS complex who remain symptomatic despite optimal medical treatment [1]. Since a substantial percentage of patients do not respond to CRT favourably, numerous studies have addressed different techniques to improve response rate [2]. These techniques imply individual optimisation of atrioventricular and interventricular delay settings, based on left ventricular (LV) filling patterns, stroke volume (SV), and asynchrony. However, despite promising single centre studies, more robust conclusions from larger trials are still needed.

Speckle tracking echocardiography (STE) has emerged as an alternative imaging tool for the evaluation of regional and global cardiac function in different clinical settings. This additive information provides new perception into cardiac physiology and thus improved patient management [3]. STE has been validated as a useful tool for the investigation of LV rotational mechanics which is a principal mechanism contributing to myocardial performance and thus SV. Considering the central role of myocardial twist in global heart function, we assumed that impaired LV twist is strongly related to asynchrony and can be restored by CRT explaining the improvement of global LV performance [4, 5].

The aim of the present study is an attempt to further explain via rotational mechanics the contribution of CRT optimisation on LV myocardial performance, by testing the distinct influence of different modes of temporal activation of ventricular electrodes (different VV delay pacing options) with parallel assessment of LV twist.

## Methods

### Study design and participants

This was a non-randomized, prospective, observational study performed in one centre. Patients indicated for a CRT implantation were consecutively included in the study. Inclusion criteria were sinus rhythm, presence of typical LBBB with QRS duration ≥ 150 ms and symptomatic HF with New York Heart Association (NYHA) functional class II to IV and LVEF ≤ 35% after optimal medical therapy. Patients were excluded if they had: (1) non-LBBB QRS morphology, (2) persistent atrial fibrillation, (3) PQ duration > 200 ms or (4) inadequate acoustic window for the ensuing echocardiography. This study was approved by the Institutional scientific board of Nikea hospital in accordance with the code of ethics of the world medical association (Declaration of Helsinki) for experiments involving humans. All patients provided informed and written consent.

### Data collection, device programming and follow up

Baseline characteristics including age, gender, body surface area (BSA), aetiology of HF, ECG, and medication were collected among all the enrolled patients.

All enrolled patients were assessed on admission by 2D-STE, and 6 minute walk test (6 min WT) and followed in the outpatient device clinic by 2D-STE (at 1 week and 6 months post-implantation) and new 6 min WT (at 6 months post-implantation) as the outcome by which to compare changes in myocardial performance and endurance activity. On their first appointment (at 1 week) all biventricular systems were optimised by changing the temporal activation of the ventricular electrodes aiming to reach the highest LV effective stroke volume (eff SV) across all activation options. On their first post implantation appointment, all patients underwent atrioventricular (AV) delay optimisation using echocardiography. Optimal AV interval was decided using mitral inflow pulsed wave Doppler based on dissociation of the E- and A-wave, with prevention of A wave truncation, and maximal AQ duration (from the end of A wave to onset of Q wave) [5]. Following AV optimisation, interventricular optimisation ensued by changing the temporal activation of ventricular electrodes. Five distinct pacing modes were sequentially attempted: exclusively right ventricular (RV) activation, initially RV followed by LV activation after 30 ms (RV–LV), simultaneous RV/LV activation, initially LV followed by RV activation after 30 ms (LV–RV) and exclusively LV activation (LV). Across all these distinct pacing options a left ventricular outflow tract (LVOT) pulsed wave interrogation was attempted with the aim of identifying the higher eff SV. Each of the selected interventricular activation modes was randomly assigned and coded by the electrophysiologist during the programming process with differing sequence. Another observer, unaware and thus unbiased for the ensuing LVOT velocity time integral (VTI) assessment performed all other imaging data analysis.

Patient response to CRT was defined as 15% improvement in LV end-systolic volume (LVESV) between the baseline and the 6-month echocardiographic assessment.

Pacemaker LV lead position implantation, determined radiographically, was classified as basal (non-optimal), midventricular (modest), or apical (optimal) [6].

### Two-dimensional and Doppler echocardiography

All patients underwent standard transthoracic echocardiography using an ultrasound machine (model ACCUSON SC2000, Siemens Healthcare, Mountain View, California) with a 1.25 to 4.5 MHz transducer (model 4V1C) and external workstation facility. A single echo operator performed all echocardiographic recordings for later analysis. Another single observer blinded to patient information and pacing status analysed off-line all imaging data. Two-dimensional, colour and spectral Doppler measurements were performed according to current guidelines [7]. The LVEF was calculated using the modified Simpson’s method [8]. Eff SV was calculated as the product of the LVOT cross-sectional area and the pulsed-wave Doppler VTI that was manually traced on the modal curve [9]. LVOT diameter was measured in parasternal long-axis view at the hinge points of the opened aortic valve cusps [10].

### Strain and rotational mechanics analysis

All speckle tracking echocardiography off-line analyses were based on initial meticulous definition of cardiac cycle time intervals. Systole was defined from the QRS onset to the instant of aortic valve closure and diastole from this time point until the ensuing QRS onset.

Two-dimensional longitudinal strain of the LV was derived from the apical four-chamber, two-chamber, and long-axis views using dedicated software installed on an external computer workstation (Siemens Healthineers eSie VVI velocity vector imaging technology, Erlangen, Germany). The echocardiographic images were recorded with temporal resolution of 60 to 70 fps. The endocardial border was manually traced, and the software automatically tracked the image speckle and produced the longitudinal strain curves in six regional segments from each apical view, respectively. A single beat was analysed, and values from three cardiac cycles were averaged.

**Fig. 1:**
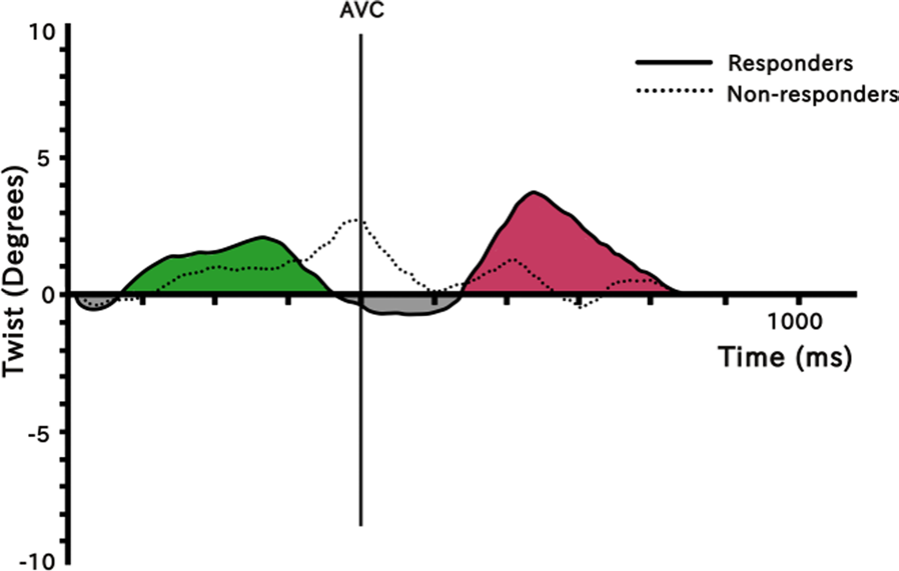
Twist curves between responders and non-responders and methodology of the Twist Integral calculation from the algebraic summation of the green (systolic) and red (diastolic) areas integrals. The negative twist values (grey color) are represented in the formula with zero number and thus excluded from the final calculation. *AVC* aortic valve closure

LV rotational deformation was calculated offline from 2-dimensional loops in the 2 parasternal basal and apical short-axis views, with temporal resolution of 60 to 70 fps. After the endocardial border was initially manually traced, the software was assisted by the user to confirm 6 basal and 4 apical segments. The basal level was recognised by the appearance of mitral leaflets while eliminating the mitral annulus, and the apical level by the presence of LV cavity in the absence of papillary muscles [11]. The 2-dimensional speckle-tracking software calculates apical and basal LV rotation from the relevant short-axis images frame by frame. Positive values were given to counterclockwise rotation and negative values to clockwise rotation when viewed from the LV apex. LV twist was defined as the absolute apex to base difference of LV rotation at isochronal time points. The following measurements were derived exclusively for subepicardial layers: peak LV systolic and diastolic twist and twist integral (TI) of the full cardiac cycle (Fig. 1). The subepicardial LV twist assessment was preferred to the other myocardial layers on the basis of being more closely related to mechanical effects of CRT [12]. For the calculation of LV TI of the full cardiac cycle, sequential isochronal apical and basal rotation measurements (twist_i_ in degrees) were exported to a worksheet Excel file (Microsoft Corporation, Redmond, WA) and the final algebraic summation (Σtwist_i _= twist_1_ + twist_2 _+$\cdots$+ twist_n_) was denoted by the formula: TΙ = systolic ΤΙ – diastolic ΤΙ (Fig. 1). The TI was further adjusted for the different duration of cardiac cycles by dividing it with the total number of discrete isochronal twist measurements (n), eliminating the impact of heart rate (Σtwist_i_/n: mean TI). Only positive twist values were included in the formula for the assessment of TI. The negative twist values during systolic or diastolic time periods were represented in the formula with a zero number since their contribution to an efficient myocardial contraction or relaxation is negligible.

### Statistical analysis

Continuous variables were expressed as mean ± standard deviation (SD) and paired Student’s t-test was used to compare the difference between baseline and follow-up in each group. Categorical variables were presented as number (percentages) by using the Pearson’s χ^2^ test or Fisher’s exact test. Only patients with available data at both enrolment, first week and 6-month appointments were included in the final analysis, and no adjustment was made for missing data. All associations among clinical and echocardiographic data were calculated using Pearson’s or Spearman’s correlation coefficient. Backward stepwise linear regression analysis was performed including all significantly related clinical and echocardiographic parameters to recognise predictors of resynchronization response in terms of at least 15% LVESV reduction. Multicollinearity in the regression analysis was examined by computation of in-model tolerance. Collinearity was considered acceptable and regression model stable for tolerance > 0.70. Repeated measures ANOVA method with Bonferroni correction was used to assess group means between responders and non-responders considering dependencies between observations within subjects in the analysis. ROC curves were generated to seek for the optimal cut-off value to predict the CRT response.

Reproducibility of eff SV and all the rotation mechanics parameters was assessed on 25 participants selected at random. Bland–Altman test and coefficient of variation (CV) were performed to evaluate the intraobserver agreement repeating the calculations 10 days later. The mean bias (Bland–Altman test) and CV for the variables eff SV, peak systolic twist, peak diastolic twist and mean TI were (a) eff SV: 0.20 ± 4.14 mL, 8%, (b) peak systolic twist: 0.00 ± 0.37°, 19%, (c) peak diastolic twist: 0.0038 ± 0.25°, 11%, and mean TI: 0.24 ± 1.72°, 21% respectively. All analyses were performed by SPSS version 25 (SPSS, Inc., Chicago, IL, USA) and a two-sided p value < 0.05 was considered statistically significant.

## Results

Of the initially enrolled 50 patients undergoing CRT, 4 were excluded from the final analysis. One was excluded due to device infection, 1 due to CRT discontinuation and 2 since were lost to follow up. Ultimately, a total of 46 consecutive patients (27 men) were included and their follow up was carried out at 6 months. Three (7%) were in NYHA functional class II, 37 (80%) in NYHA III and 6 (13%) in NYHA IV respectively. All patients were on stable, optimal HF medical therapy according to current guidelines. In 33 patients HF had non-ischemic aetiology and the remainder (13 patients) had a history of coronary artery disease. The mean LVEF was 29.2 ± 7.1%, and the mean QRS duration was 173 ± 20 ms.

Twenty-two (48%) patients were responders at 6-month follow-up and most of them had dilated cardiomyopathy. Baseline clinical and echocardiographic characteristics of responders versus non-responders are summarized in Table 1. There were no differences in baseline characteristics between responders and non-responders, except for HF aetiology and LVEF. QRS duration of responders was also marginally higher, yet with a trend to statistical significance.

**Table 1:** Baseline clinical and echocardiographic characteristics of CRT responders vs. non-responders

Variable	CRT responders [22]	CRT non responders [24]	*P* value
Age	64 ± 10	66 ± 7	0.544
Male	13 (48%)	14 (52%)	0.958
NYHA functional class (II/III/IV)	3/16/3	0/21/3	0.166
Ischemic etiology	1 (4%)	12 (50%)	0.001
Non-ischemic etiology	21 (96%)	12 (50%)	0.001
Hypertension	10 (46%)	14 (58%)	0.382
Mean arterial pressure (mmHg)	97 ± 10	91 ± 13	0.113
Heart rate (bpm)	75 ± 11	69 ± 11	0.102
Creatinine (mg/dL)	1.1 ± 0.4	1.1 ± 0.2	0.570
QRS duration (ms)	179 ± 21	168 ± 18	0.060
6 min walk test (m)	166 ± 90	199 ± 72	0.179
Medication			
ACE inhibitors or ARBs or ARNI	22 (100%)	24 (100%)	–
β-Blockers	16 (73%)	19 (79%)	0.609
Diuretics	19 (86%)	20 (83%)	0.775
MRAs	18 (82%)	21 (88%)	0.592
SGLT2 inhibitors	6 (27%)	8 (33%)	0.754
Baseline echocardiography			
Mitral regurgitation grade III or IV	8 (36%)	8 (33%)	0.829
LVEF (%)	31 ± 7	27 ± 7	0.048
EDV (mL)	215 ± 49	239 ± 56	0.139
ESV (mL)	151 ± 49	175 ± 52	0.109
Eff SV (mL)	55 ± 14	54 ± 14	0.747
GLS (%)	− 10 ± − 2	− 8 ± − 3	0.107
Peak twist in systole (°)	1.34 ± 1.20	1.73 ± 1.13	0.203
Peak twist in diastole (°)	2.38 ± 1.28	1.46 ± 1.07	0.035
TI (°)	− 16.34 ± 11.19	0.53 ± 10.59	< 0.001
Mean TI (°)	− 0.34 ± 0.23	0.004 ± 0.22	< 0.001

*ACE* angiotensin converting enzyme, *ARBs* angiotensin receptor blockers, *ARNi* angiotensin receptor–neprilysin inhibitor, *EDV* end diastolic volume, *ESV* end systolic volume, *GLS* global longitudinal strain, *LV* left ventricular, *LVEF* LV ejection fraction, *MRAs* mineralocorticoid receptor antagonist, *SGLT2* sodium–glucose cotransporter 2 inhibitors, *TI* twist integral, *6 min WT* 6 min walk test

The LV lead was optimally located in almost all responders (basal, midventricular and apical position: 0/1/21 patients) vs. non-responders (3/8/13 respectively, p: 0.006). The commonest selected interventricular activation mode was the simultaneous activation of the 2 ventricular leads (39% RV/LV; 7 responders vs. 11 non-responders), followed equally by RVLV (24%; 3 responders vs. 8 non-responders) and LVRV modes (24%; 7 responders vs. 4 non-responders). Interestingly in 6 patients (13%) the optimal eff SV was achieved by LV lead activation only (5 responders vs. 1 non-responder). On the contrary, single RV lead activation was never selected since the provided eff SV was always lower than other pacing options.

Table 2 shows the associations between demographic, clinical and echocardiographic parameters and the final improvement in LVESV. The strongest associations were noticed in the mean TI at admission, the mean TI at 1 week, the max systolic twist at 1 week and the etiology of HF, followed by pre-ejection and ejection time of the RV contraction. Interestingly, the difference (Δ) of several other variables between the first 2 visits of the patients was also associated with LVESV improvement. In particular, Δ mean TI, Δ systolic twist, Δ eff SV, Δ GLS and Δ QRS showed significant relationships.

**Table 2:** Relationship and independent predictors among improved LVESV response and demographic, clinical and echocardiographic parameters

Variable	*r*	*p* value
**Clinical**		
BSA	− 0.270	0.069
Ischemic etiology of HF	− 0.514	< 0.001
QRS duration	0.261	0.079
QRS difference	0.464	0.0011
HR on admission	− 0.270	0.070
HR at 1 week	− 0.306	0.039
Activation mode	0.343	0.020
Position of electrode implantation	0.306	0.038
**Echocardiographic**		
LV filling time	0.247	0.098
RV preejection time	− 0.358	0.015
RV ejection time	0.396	0.006
Peak twist during diastole on admission	0.318	0.019
Mean TI	− 0.639	< 0.001
Peak twist during systole at 1 week	0.665	< 0.001
Mean TI at 1 week	0.707	< 0.001
Difference (Δ) in eff SV	0.646	< 0.001
Δ GLS	− 0.324	0.028
Δ Peak systolic twist	0.635	< 0.001
Δ Max diastolic twist	− 0.240	0.113
Δ Mean TI	0.797	< 0.001

Variable	β	p value
**Linear regression analysis**		
Difference (Δ) in eff SV	0.410	< 0.001
Δ mean TI	0.265	0.007
Ischemic etiology of HF	− 0.242	0.002
Δ Peak systolic twist	0.219	0.013
RV preejection time	− 0.174	0.015
Position of electrode implantation	0.169	0.002

*BSA* body surface area, *GLS* global longitudinal strain, *HF* heart failure, *HR* heart rate, *LV* left ventricular, *RV* right ventricular, *TI* twist integral, *eff SV* effective stroke volume

In the subsequent regression analysis (Table 2), all significantly associated variables were included as potential predictors of ensuing improvement in LVESV at 6 months. The strongest predictor of LVESV improvement was the change of eff SV between admission and first appointment at clinic, followed by the change of the mean TI and the difference of systolic max twist. Other independent predictors were the apical position of the LV electrode and the short RV preejection time, a finding representing the preserved inotropic state of the RV.

**Fig. 2:**
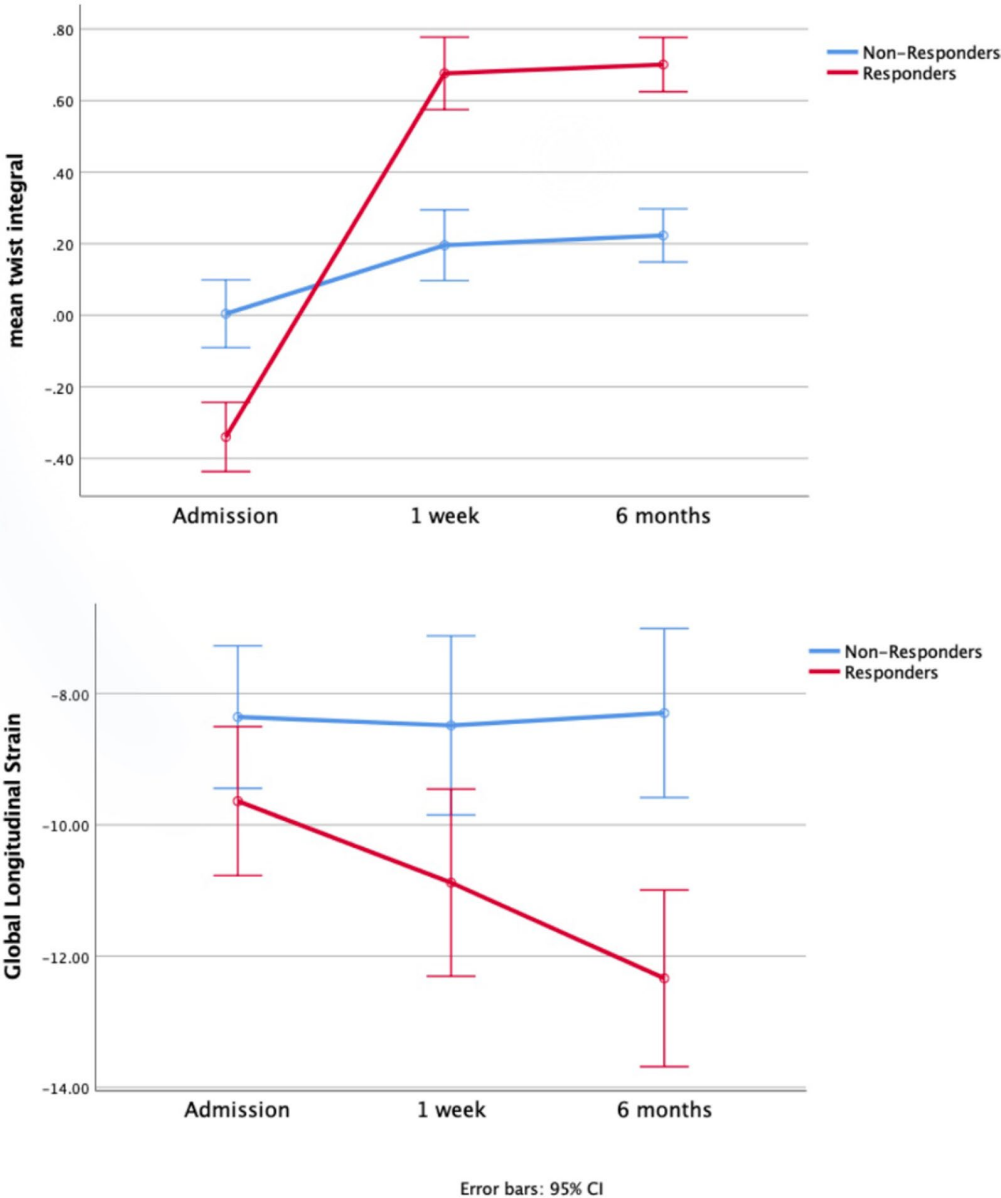
Sequential change of mean TI and GLS during the study period

Table 3 and Fig. 2 demonstrate the sequential change of several twist-based variables during the 3 appointments of the study patients. Interestingly, responders showed an immediate and continuous improvement of all LV rotational mechanics parameters for at least 6 months after CRT, though a similar but weaker increase was also detected in mean TI variable in non-responders. However, in non-responders no significant serial changes were detected in both peak systolic and diastolic twist parameters. In agreement with the previous observations, though the exercise tolerance between the 2 groups was comparable at admission (Table 1), the responders also improved baseline walk at 6 months (responders vs. non-responders 273 ± 113 vs. − 12 ± 75 *p* > 0.001 respectively).

**Table 3:** Sequential change of twist variables during the study period

Echocardiography variable	Admission	1st appointment	6 months
Non-responders			
Peak twist in systole	1.73 ± 1.13	2.01 ± 1.11	1.84 ± 1.00
Peak twist in diastole	1.46 ± 1.07	1.31 ± 1.10	1.06 ± 0.71
TI	0.53 ± 10.59	9.36 ± 12.09***	10.41 ± 6.85***
Mean TI	0.004 ± 0.22	0.20 ± 0.25***	0.22 ± 0.15***
Responders			
Peak twist in systole	1.34 ± 1.20	3.86 ± 1.00***	3.95 ± 1.10***
Peak twist in diastole	2.38 ± 1.28	1.11 ± 0.93**	1.25 ± 0.85*
TI	− 16.34 ± 11.19	29.96 ± 8.47***	33.76 ± 9.44***
Mean TI	− 0.34 ± 0.23	0.67 ± 0.21***	0.70 ± 0.20***

Multiple post hoc tests comparing follow-up with admission values. No significant serial changes were noted between 1 appointment and 6 months. *p < 0.05, **p < 0.01, ***p < 0.001

**Fig. 3:**
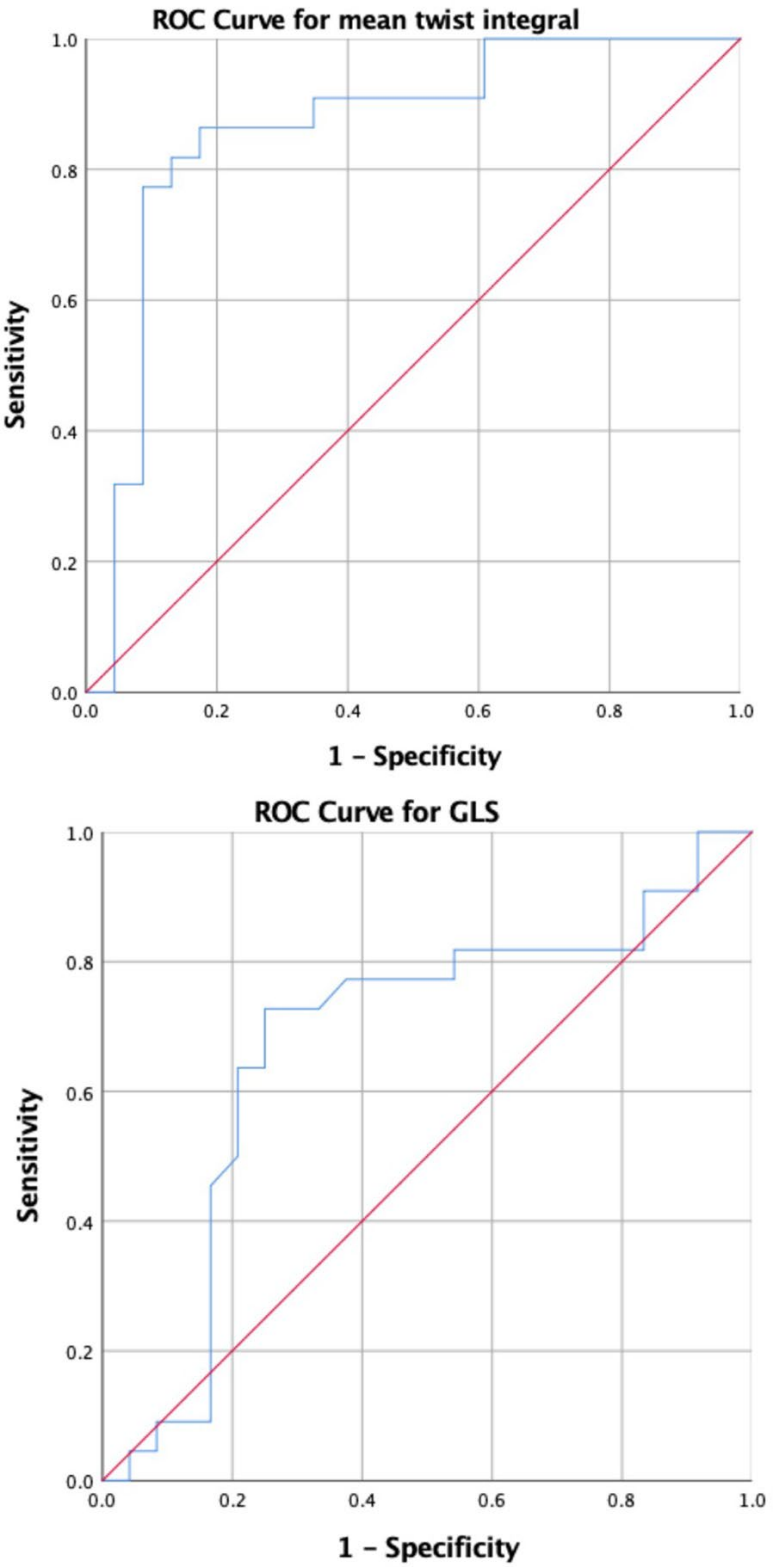
ROC curves for mean TI and GLS

The mean TI showed a better performance than GLS (Fig. 3) for the prediction of CRT response (areas under the curve 86.2%; *p* < 0.001 and 67.4%; p: NS respectively). A mean TI value of − 0.11° showed the greatest diagnostic accuracy to predict improvement (sensitivity 86.4% and specificity 83.6%), while GLS was considered weak as a predictor. The best GLS value to predict response to CRT was − 9.15% with a sensitivity of 72.7% and a specificity of 75%.

## Discussion

The present study provides two main conclusions that may be used as hypotheses generating for further research. Firstly, the implementation of a new strategy for CRT optimisation via adjusted temporal activation of the ventricular pacemaker electrodes (guided by the higher effective LV SV) proved to be beneficial for LV reverse remodeling in line with improved exercise tolerance of the responders. Secondly, we attempted to explain the mechanism of this therapeutic maneuver through the improved rotational mechanics of the failing LV. It was pointed out that beyond AV optimisation, different temporal activation of the pacing leads can further impact on the effective LV contraction via improved LV twist.

The additive contribution of cardiac imaging to detect which patients with HF benefit from CRT has been investigated in numerous studies still with insufficient conclusions [1, 13–16]. The only robust data implemented in the current guidelines as preimplantation criteria and thus requested by the clinician are clinical status, QRS assessment and LVEF. No other echocardiographic or other imaging data of asynchrony is considered essential before CRT [1].

Assessment of LV mechanical asynchrony may provide an additive contribution in patients undergoing CRT. Several studies encourage the concept that it may predict CRT response or at least support CRT indication. However, it is still uncertain what is the best echocardiographic index of cardiac asynchrony, although recently STE and 3D echocardiography have provided reliable and more predictive data than those used prior to these innovative imaging modalities [17]. LV myocardial torsional mechanics are known to be compromised in HF patients [18–20], though an increasing interest for its role in assessing improvement to CRT is recently noticed [21]. The reasons of this investigating hesitancy might be the low reproducibility of measurements, the complex helical ventricular architecture and contraction and the different vectors’ software tools that frequently provide contradictory data of identical phenomena.

The 2 study groups were not comparable in all their baseline clinical and echocardiographic characteristics. As anticipated, the responders were primarily patients with non-ischemic cardiomyopathy with their QRS complex being borderline wider than non-responders. The concomitant medical therapy was optimal in the two groups except for a low percentage of SGLT2 inhibitors usage since the enrolment of the patients completed before the advent of the updated HF guidelines that propagated this therapy [22]. Regarding the echocardiographic data, the LVEF of the responders was higher while their GLS showed a relevant trend for improved LV contractility on admission. STE revealed higher values of peak twist in diastole in responders, a finding that reveals the suspended LV contraction after aortic valve closure and thus the beneficial contribution of CRT therapy to restore this deleterious contractile abnormality. Interestingly, the novel echocardiography variable of TI was remarkably different between the 2 groups, comprising in one number both the contractile systolic and diastolic ventricular twist elements of the cardiac cycle. The finding of higher admission GLS values in responders can be explained by the fact that several myocardial segments may reach their peak strain in asynchronous cardiac contraction in the diastolic period of the cardiac cycle something that is not usually well apprehended in post study automatic analysis report.

Linear regression analysis revealed which of the numerous investigated variables can independently predict a beneficial CRT response. The higher contribution was shown by the improvement of both the eff SV and mean TI. Moreover, variables such as non-ischemic CMP as a HF etiology, the improvement of peak systolic twist between admission and the first appointment after system optimisation, the RV pre-ejection time and the proper implantation of LV electrode were independently remained valuable predictors of improvement.

The short RV prejection time as criterion of good inotropic state supports the clinical assumption that the functional integrity of the RV should not be overlooked before implantation of biventricular pacing systems.

In this study we enrolled patients with QRS duration ≥ 150 ms, since are more likely to respond favourably (class I, level A) than the subgroup of 130–129 ms (class IIa, level B) which demonstrates not so strong evidence of CRT effectiveness. Importantly, though QRS complex duration improvement showed an association with the response, this variable was excluded in the ensuing multivariable analysis. This result might be attributed to the strong predictive value of the novel echocardiography index (mean TI) that weakened the contribution of other parameters in the prediction model.

Several parameters of LV twist were evaluated with the intention to interpret the beneficial mechanism of CRT. The predicted response might be heralded by lower baseline peak systolic than diastolic twist and negative TI values, findings that depict the harmful twisting of the LV after the aortic valve closure (Fig. 1). The post CRT modification of all the LV rotational mechanics parameters in responders, with improved twist in systole and less harmful (positive) twist in diastole, partly explains the beneficial contribution of resynchronization therapy at least in these patients. Interestingly, mean TI showed a weaker but still significant change in non-responders (Table 3; Fig. 2). This finding should highlight the current recommendation that until more robust data from future trials no patient should be denied CRT based upon echocardiography [23]. Even patients who fail to demonstrate reverse remodeling and require a heart failure admission, still derive haemodynamic benefit from CRT, as they often deteriorate when biventricular pacing is temporarily stopped [24]. It needs to be emphasized that if CRT is being implanted in HFrEF patients with a QRS width *>* 130 ms (especially in the presence of LBBB), there is no recognized patient population that experiences a negative response to CRT [25].

## Limitations

This study was designed to investigate whether the assessment of rotational mechanics via a new proposed index can be utilised as potential predictor for patients treated with CRT. Because of the open, non-randomised type of the study, which was only hypothesis-generating, more randomized clinical prospective trials are encouraged to establish LV twist as a robust step of the screening process for the HF patients for more effective CRT. We also demonstrated a good intraobserver agreement for effective SV (a crucial step in the study workflow) and a weaker but acceptable agreement for all other parameters of rotation mechanics assessment. Still, these results represent just the post examination calculations in the workstation and not the previous step of either the LVOT pulse Doppler interrogation or the demanding numerous 2D acquisitions. It is well known the variability of measurements with just a minor movement of the cursor or the transducer especially in long lasting and demanding echocardiographic studies.

## Conclusions

The results of the present study offer evidence about the importance of biventricular on top of AV optimisation for post-CRT patients. It was also pointed out that rotation mechanics assessment may be used as an additive tool for the better initial discrimination of candidates for effective CRT. The findings also underscore that the success of biventricular pacing may also be explained by the rotation mechanics improvement not only during systole but also via the attenuation of the deleterious contribution of LV twist in diastole. However, these conclusions need further confirmation from larger clinical trials.

## Data Availability

No datasets were generated or analysed during the current study.

## Abbreviations

AV: Atrioventricular
BSA: Body surface area
CRT: Cardiac resynchronization therapy
CV: Coefficient of variation
eff SV: Effective stroke volume
ESV: End-systolic volume
GLS: Global longitudinal strain
HF: Heart failure
HFrEF: Heart failure with reduced ejection fraction
LV: Left ventricular
LVEF: Left ventricular ejection fraction
LVOT: Left ventricular outflow tract
RV: Right ventricular
SD: Standard deviation
STE: Speckle tracking echocardiography
SV: Stroke volume
TI: Twist integral
VTI: Velocity time integral
6 min WT: Six minute walk test
